# Benchmarking in vitro tissue-engineered blood–brain barrier models

**DOI:** 10.1186/s12987-018-0117-2

**Published:** 2018-12-04

**Authors:** Jackson G. DeStefano, John J. Jamieson, Raleigh M. Linville, Peter C. Searson

**Affiliations:** 1 Institute for Nanobiotechnology, Johns Hopkins University, Baltimore, MD USA; 2 Department of Materials Science and Engineering, Johns Hopkins University, Baltimore, MD USA; 3 Department of Chemical and Biomolecular Engineering, Johns Hopkins University, Baltimore, MD USA; 4 Department of Biomedical Engineering, Johns Hopkins University School of Medicine, Baltimore, MD USA; 5 120 Croft Hall, Johns Hopkins University, 3400 North Charles Street, Baltimore, MD 21218 USA

**Keywords:** Blood–brain barrier, Tissue-engineering, Induced pluripotent stem cells, Benchmarking, In vitro modeling, Brain microvascular endothelial cells

## Abstract

The blood–brain barrier (BBB) plays a key role in regulating transport into and out of the brain. With increasing interest in the role of the BBB in health and disease, there have been significant advances in the development of in vitro models. The value of these models to the research community is critically dependent on recapitulating characteristics of the BBB in humans or animal models. However, benchmarking in vitro models is surprisingly difficult since much of our knowledge of the structure and function of the BBB comes from in vitro studies. Here we describe a set of parameters that we consider a starting point for benchmarking and validation. These parameters are associated with structure (ultrastructure, wall shear stress, geometry), microenvironment (basement membrane and extracellular matrix), barrier function (transendothelial electrical resistance, permeability, efflux transport), cell function (expression of BBB markers, turnover), and co-culture with other cell types (astrocytes and pericytes). In suggesting benchmarks, we rely primarily on imaging or direct measurements in humans and animal models.

## Introduction

Recent advances in stem cell technology, tissue engineering, and microfluidics have led to rapid advances in the complexity of in vitro models of the blood–brain barrier (BBB). Stem cell technology provides a reliable source of human, brain-specific cells: iPSC-derived human brain microvascular endothelial cells (dhBMECs) exhibit many of the hallmarks of human BMECs [1–29], a long-standing problem in developing BBB models [30–32]. Additionally, protocols for iPSC-derived astrocytes, pericytes, microglia and neurons have been developed to facilitate modeling of the neurovascular unit [33]. Similarly, advances in tissue engineering and microfluidics provide the tools for organization of perfusable microvessels or microvascular networks [34, 35]. Diverse BBB-on-a-chip models have emerged over the last 5 years, they can generally be classified as: (1) two-dimensional microfluidic models, (2) hybrid microfluidic models, (3) three-dimensional templated models or (4) self-organization models. Two-dimensional microfluidic models incorporating a permeable membrane (resembling that of a traditional Transwell^®^ assay) are extremely valuable for applications such as drug screening or measures of electrical resistance, however, these models do not recapitulate many aspects of physiological BBB function [36–39]. Hybrid microfluidic models capture more complexity but lack homogenous cell-ECM interactions and cylindrical geometry and are thus not able to respond to vasodilation/constriction [40, 41]. Templating approaches support generation of singular cylindrical microvessels embedded within an extracellular matrix that can be integrated into a flow system for live-cell imaging [23, 24, 42–44]. Lastly, self-organization approaches that mimic vasculogenesis and/or angiogenesis have emerged to generate multicellular models of brain microvascular networks [45, 46]. Since animal models do not always recapitulate human physiology or disease [47, 48], in vitro models can provide an important link between human physiology and animal models.

The value of BBB models in basic and translational research is dependent on the ability to recapitulate in vivo and ex vivo studies. The fidelity of the model is usually dictated by the purpose and the processes under study. More reductive models will naturally recapitulate fewer characteristics of the BBB, while more complex models attempt to recapitulate more characteristics but are usually lower throughput. In all cases, benchmarking to in vivo studies is key to establishing physiological relevance.

Benchmarking is surprisingly challenging, in large part because much of our knowledge about BBB structure and function is derived from in vitro studies. Here we describe 12 design criteria for tissue engineering the human BBB. This is not intended to be a complete checklist of benchmarks for model validation, but a limited set of parameters associated with structure (ultrastructure, wall shear stress, geometry), microenvironment (basement membrane and extracellular matrix), barrier function [transendothelial electrical resistance (TEER), permeability, efflux transport], cell function (expression of BBB markers, turnover), and co-culture with other cell types (astrocytes and pericytes). Depending on the purpose of the model, the specific benchmarks may vary and may not include all those listed here. Wherever possible, benchmarks are suggested based on imaging or direct measurement in humans or animal models.

## Benchmarks for blood–brain barrier models

### Ultrastructure

**Fig. 1:**
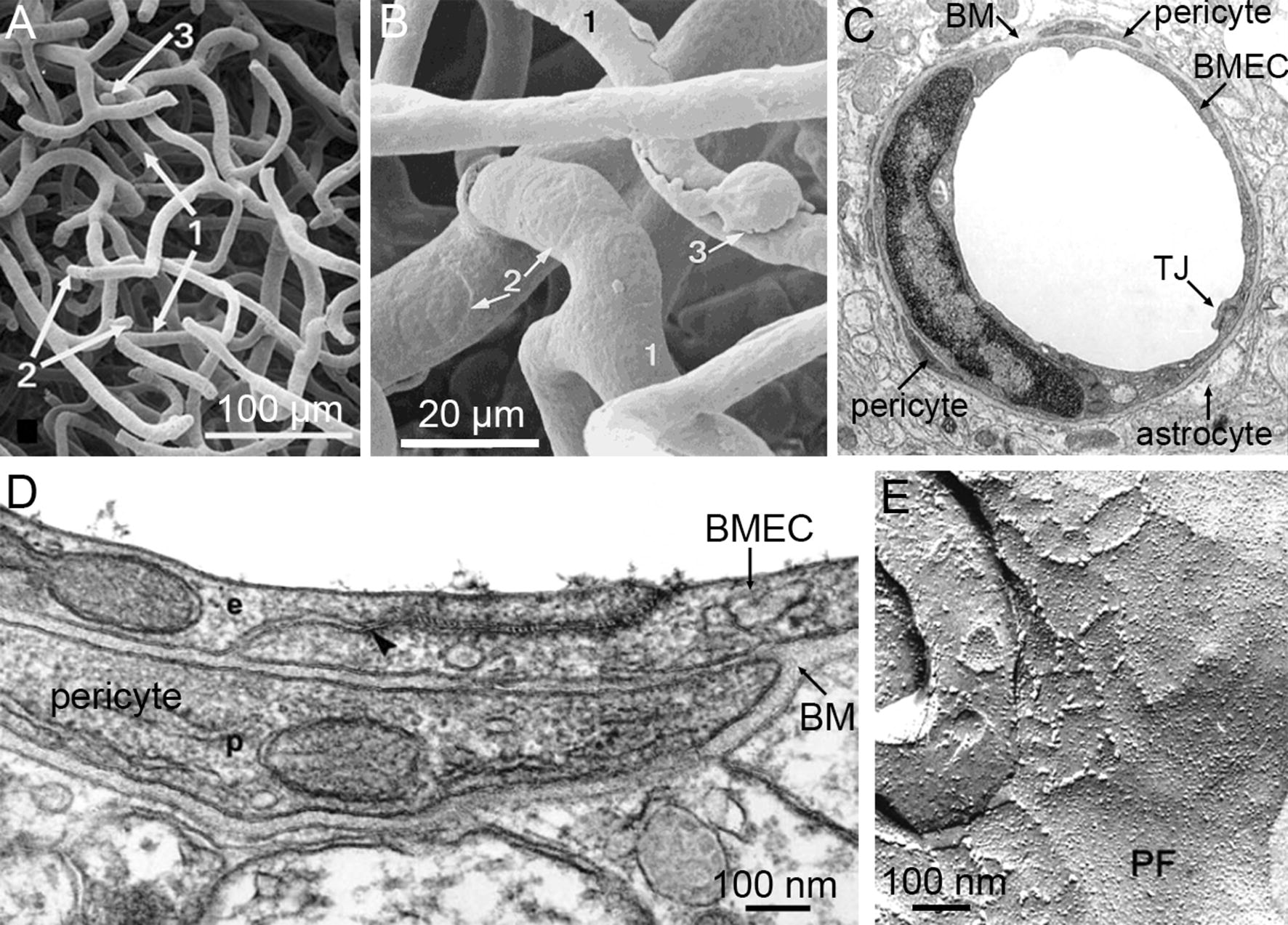
EM images of brain microvessels. **A** Scanning electron micrograph of a replica of cortical capillaries in the frontal lobe of the human brain (from [74]). **B** Scanning electron micrograph of a replica of a cortical capillary network in the human brain showing the imprint of endothelial cell nuclei (2) and a pericyte with a “bump on a log” morphology (3) (from [74]). **C** Cross-section of a capillary in the frontoparietal cortex of a Wistar–Kyoto rat showing an endothelial cell wrapping around to form a tight junction with itself, along with associated pericytes and astrocyte end-feet (from [70]). **D** Transmission electron microscope cross-section of a cortical capillary in a rat. A systemically injected lanthanum compound penetrated the inter-endothelial space up to the tight junction (from [71]). **E** Freeze-fracture replicas of cerebral endothelial cell tight junctions. In capillary preparations, most P-face strands are occupied with particles (from [75])

To power the adult human brain, nutrients are supplied to the 100 billion neurons via a 600 km network of capillaries and microvessels [49]. Since the brain does not have significant capacity to store metabolic nutrients, cerebral blood flow is proportional to cerebral metabolic rate [50], and the cell bodies of neurons are typically 10–20 µm from the nearest capillary [51, 52]. Capillaries are supplied by arterioles and drained by post-capillary venules which are up to 100–200 µm in diameter. Capillaries in the human brain are 8–10 µm in diameter, with 50–100 µm long segments between bifurcations (Fig. 1A, B) [53–56]. In contrast, the smallest capillaries in the mouse brain are around 3 µm in diameter, and in the rat brain are around 4 µm in diameter [57]. In capillaries, BMECs wrap around to form junctions with themselves and their upstream and downstream neighbors (Fig. 1C, D). The spatial arrangement of pericytes and astrocytes are described in subsequent sections. Post-capillary venules (PCVs) are characterized by a perivascular space with limited supporting cells [58–60]. Evidence suggests that extravasation of leukocytes, tumor cells, and parasites occurs preferentially at PCVs [60–66].

Electron microscopy (EM) images of the ultrastructure of microvessels and capillaries in rodent brains show BMECs are relatively flat and have considerable cell–cell overlap (typically 0.5 µm or more) (Fig. 1C), likely associated with tight junction formation [67–71]. In EM images of the overlapping regions using freeze-fracture, tight junctions appear as a network of contact points or particles between the extracellular domains of claudin-5, occludin and other transmembrane proteins on opposing membranes (Fig. 1E) [72, 73].

In establishing BBB models, desired geometry is dictated by the diameter and location of the target microvessel (arteriole, capillary, or venule), which also determines other aspects of the local microenvironment (discussed in subsequent sections).

### Wall shear stress

Wall shear stress is thought to regulate many processes associated with the endothelium [76–79], however, much of our knowledge comes from in vitro experiments where the characteristics of the BMECs and the 2D geometry may influence results. Nonetheless, shear stress can play a role in mediating processes such as leukocyte adhesion, where the probability of capture is higher in post-capillary venules where the shear stress is relatively low. In 3D models, wall shear stress can be determined from particle image velocimetry (PIV) via analysis of fluorescent beads in the perfusion media or by calculating flow rate. These methods supports determination of the flow profile and wall shear stress within a microfluidic device or tissue-engineered microvessel [76, 77, 80]. Wall shear stress can also be estimated from the velocity of red blood cells (RBCs) which enables correlation with in vivo experiments.

In the arterial tree, the wall shear stress is 10–70 dyne cm^−2^ depending on vessel diameter [81, 82]. Fluctuations in blood pressure during the cardiac cycle, typically oscillating between 80 and 120 mmHg, lead to pulsatile arterial blood flow [83]. These fluctuations are damped as the vessel diameter decreases, resulting in near constant blood flow in small arterioles, capillaries and venules. RBC velocity in brain capillaries in rodent models is in the range of 0.5–2.0 mm s^−1^ [84–91], corresponding to a wall shear stress of about 20–40 dyne cm^−2^ [92]. The large range of shear stress in capillaries is in part due to neurovascular coupling and the wide range of metabolic demands [93–98]. In mouse post-capillary venules (PCVs), the flow is highly damped with an average RBC velocity of 5–7 mm s^−1^ corresponding to an average wall shear stress of 1–4 dyne cm^−2^ [84, 92, 99–101]. Erythrocyte velocity was used to estimate a mean wall shear stress of 1–6 dyne cm^−2^ in capillaries and PCVs ranging in size from 4 to 20 µm in the human bulbar conjunctiva [99].

In benchmarking in vitro models, the flow system should be designed to achieve the average wall shear stress of the microvessel type (arteriole, capillary, or venule). For the case of arterioles and capillaries, recapitulating pulsatile flow may also be important as it has been shown to influence barrier function, solute transport along the endothelium and inflammation within in vitro BBB models [44, 79, 102].

### Cylindrical geometry

The cylindrical geometry of microvessels imposes two important constraints on BMECs. First, cells experience curvature, which is inversely related to diameter, and plays a role in BMEC behavior. Human BMECs in confluent monolayers resist elongation and alignment due to curvature, whereas other ECs elongate and align to minimize the effects of curvature [103]. This may represent an evolutionary advantage by reducing the total length of cell–cell junctions per unit length of vessel, thereby reducing paracellular transport. Second, the cylindrical geometry means that there is a finite number of cells around the perimeter of a microvessel. In capillaries where BMECs wrap around and form tight junctions with themselves, there is one cell around the perimeter. In microvessels there are relatively few cells around the perimeter and hence cell–cell interactions and processes such as motility are extremely limited in comparison to 2D monolayers [24, 103]. Whether cylindrical geometry and shear stress are critical to achieve physiological barrier function is not well understood. However, permeabilities for Lucifer yellow in iPSC-derived human BMEC microvessels and in the Transwell^®^ assay are similar [24], suggesting that physiological geometry and shear stress are not prerequisites for tight junction formation.

### Basement membrane

The basement membrane surrounding the endothelial cells in the cerebrovasculature consists of fibronectin, laminin, collagen type IV, heparan sulfate proteoglycans (HSPG) such as perlecan, and nidogens/entactins [57, 104–108]. The thickness (20–200 nm) and composition of the basement membrane is dependent on the location in the cerebrovasculature. In arteries and arterioles the presence of perivascular smooth muscle cells results in an inner endothelial basement membrane and an outer the parenchymal basement membrane, in which distinct laminin isoforms are present [109]. In capillaries, the basement membrane is relatively thin and occupies the space between the endothelial cells and astrocyte end-feet, and surrounds pericytes (see Fig. 1C, D) [71, 110]. In PCVs, the endothelial and parenchymal basement membrane are separated by the perivascular space. The basement membrane in capillaries is typically characterized by the presence of laminin α4 and α5. In tissue-engineered models, basement membrane proteins can be deposited before cell seeding to promote adhesion of cells [23]; cells will then modify their local environment as they become established and secrete additional basement membrane proteins.

### Extracellular matrix (ECM)

Selection of an extracellular matrix material is one of the major challenges in developing tissue-engineered models of the BBB since 70–85% of the brain volume is cells [111]. The extracellular space consists of a hyaluronic acid-based extracellular matrix and brain interstitial fluid. The extracellular space is characterized by an interconnected network of pores, 50–100 nm in size that serve as a reservoir for ions and a pathway for transport [111–114]. The extracellular volume fluctuates during normal brain function and decreases during development and aging [111, 115]. The extracellular matrix in the interstitial space is composed of hyaluronic acid (HA), lecticans (aggrecan, versican, neurocan, and brevican), hyaluronan and proteoglycan link proteins (HAPLNs), and tenascins [116, 117]. Common ECM proteins such as collagen type I and fibronectin are not present in the healthy brain [118]. As a result, tissue engineered models of the BBB must either incorporate large numbers of neurons and astrocytes, as well as other glial cells, or use a passive matrix material that provides structural support for BBB microvessels (see “Astrocyte” section for more details).

### Expression of BBB markers

**Fig. 2:**
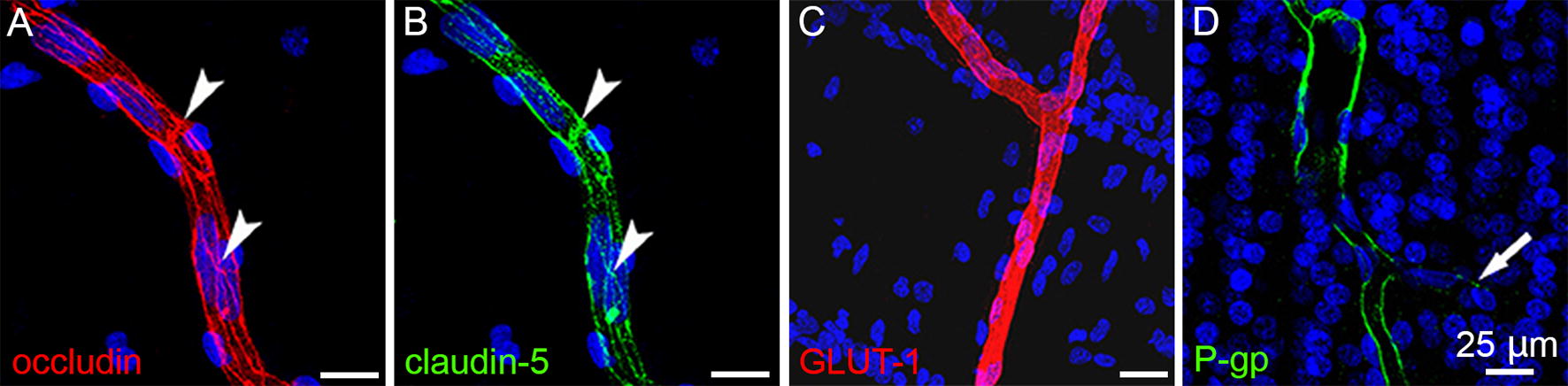
Confocal images of key BBB markers stained in human cerebral cortex at mid-gestation tissue sections (from [126]). **A**, **B** Occludin and claudin-5 display clear junctional distributions with individual endothelial cells wrapping around to form junctions with themselves and neighboring cells. **C**, **D** GLUT-1 and P-gp, critical nutrient and efflux transporters, display uniform expression

The expression of tight junctions (TJs) and transport systems are widely used to characterize in vitro BBB models [32]. Tight junctions between adjacent BMECs minimize paracellular permeability, maintain cell membrane polarization, and facilitate intracellular signaling [119]. Tight junctions are comprised of transmembrane proteins (i.e. occludin, claudins, junctional adhesion molecules) that interact with cytoplasmic scaffolding proteins (i.e. zona occludens), the actin cytoskeleton, and associated signaling proteins [120]. While many cell types express tight junctions, claudin-5 is endothelial-cell specific and particularly enriched in the brain cerebrovasculature compared to other TJ components [121, 122]. Localization of claudin-5, occludin, junctional adhesion molecules, and zona occludens-1 are commonly used to validate cell sources for in vitro models [1, 2, 4, 19, 32, 123, 124]. Immunocytochemistry can be used to visualize these proteins at cell–cell junctions; importantly, TJ strands should be crisp (Fig. 2A, B), while under pathological or non-physiological conditions, they are non-continuous or show intracellular localization [7, 12, 14, 125].

Additionally, the brain endothelium is enriched in nutrient and efflux transporter systems. In the brain, Glucose transporter 1 (GLUT1) is highly expressed only in endothelial cells, and hence is a common biomarker for brain microvessels and capillaries (Fig. 2C). GLUT1 expression is critical to facilitate transport of glucose to meet the high metabolic demand of the brain. The P-glycoprotein (P-gp) efflux pump is one of several multi-spectrum efflux transporters present in brain endothelium and is predominantly localized to the luminal membrane. Expression of the P-gp pump verifies the potential for efflux, an important component of the BBB barrier function (see “Efflux transport” section for more details).

A minimum set of criteria for staining in vitro BBB models is: Continuous TJ proteins (i.e. claudin-5, occludin, and zona occludens-1) localized to cell–cell junctions, and uniform expression of nutrient and efflux transporters.

### Transendothelial electrical resistance (TEER)

**Fig. 3:**
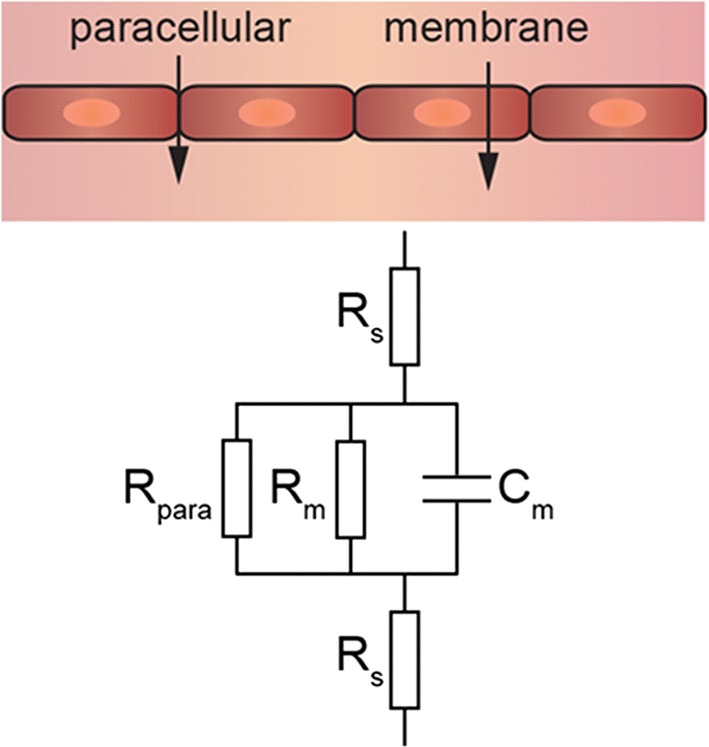
Schematic illustration of the equivalent circuit for the impedance of a confluent monolayer. R_para_—resistance associated with paracellular ion transport, R_m_—resistance associated with ion transport across the cell membrane (i.e. through ion channels), C_m_—capacitance associated with the cell membranes, R_s_—resistance associated with the media. If R_para_ > R_m_ then paracellular ion transport is negligible and TEER values are determined by the resistance (1/conductance) of the endothelial cell membranes

TEER measures the ionic resistance of cell monolayers. The equivalent circuit for endothelial and epithelial monolayers has two components, the resistance associated with paracellular ion transport and the resistance associated with ion transport across the apical and luminal cell membranes (Fig. 3) [57]. For the limiting case where paracellular resistance is large, implying negligible paracellular permeability, then TEER values are determined by the conductance of the cell membranes. TEER is difficult to measure in tissue-engineered microvessels since there is usually a low resistance pathway between the lumen and surrounding matrix at the entry and exit. One solution to this problem is to measure both TEER and the permeability of a small molecule (e.g. Lucifer yellow) in a transwell assay, and the permeability of the small molecule in microvessels. If the small molecule permeability is the same in 2D and 3D, and the TEER value in 2D is in the physiological range, then this implies that the tissue engineered model has physiological TEER. However, this method should be used with caution: although TEER is approximately inversely related to permeability, the relationship is non-linear and dependent on the endothelial cell type and solute [5, 127]. For example, iPSC-derived hBMECs with TEER above 900 Ω cm^2^ display a constant IgG permeability, indicating this critical cutoff for physiological studies of large molecule transport [5].

Measurements in pial and arterial microvessels in rats and pial microvessels in frogs have shown TEER values in the range 1500–6000 Ω cm^2^ [128–130]. Calculations based on the conductance and density of ion channels predict BMEC electrical resistance of 4000–8000 Ω cm^2^ [128, 131]. In contrast, TEER for primary and immortalized BMECs in monoculture are typically ≤ 200 Ω cm^2^ [132–134]. The TEER of iPSC-derived BMECs are typically ≥ 1500 Ω cm^−2^, within the range obtained for microvessels in animal models [1, 2, 4, 5]. An important implication of the observation that physiological TEER is achieved with iPSC-derived BMECs in 2D, is that cylindrical geometry, shear flow and co-culture are not essential for the tight junction formation.

### Permeability

Tight junctions effectively block paracellular transport and hence small molecules cross the normal BBB either by passive diffusion or by carrier- or receptor-mediated transport. Downregulation of tight junctions can lead to disruption of the BBB and the onset of paracellular transport. Since many primary and immortalized cell lines do not exhibit physiological TEER, it is likely that the permeability values measured in Transwell^®^ experiments utilizing these cells reflect contributions from both paracellular and transcellular transport pathways.

**Fig. 4:**
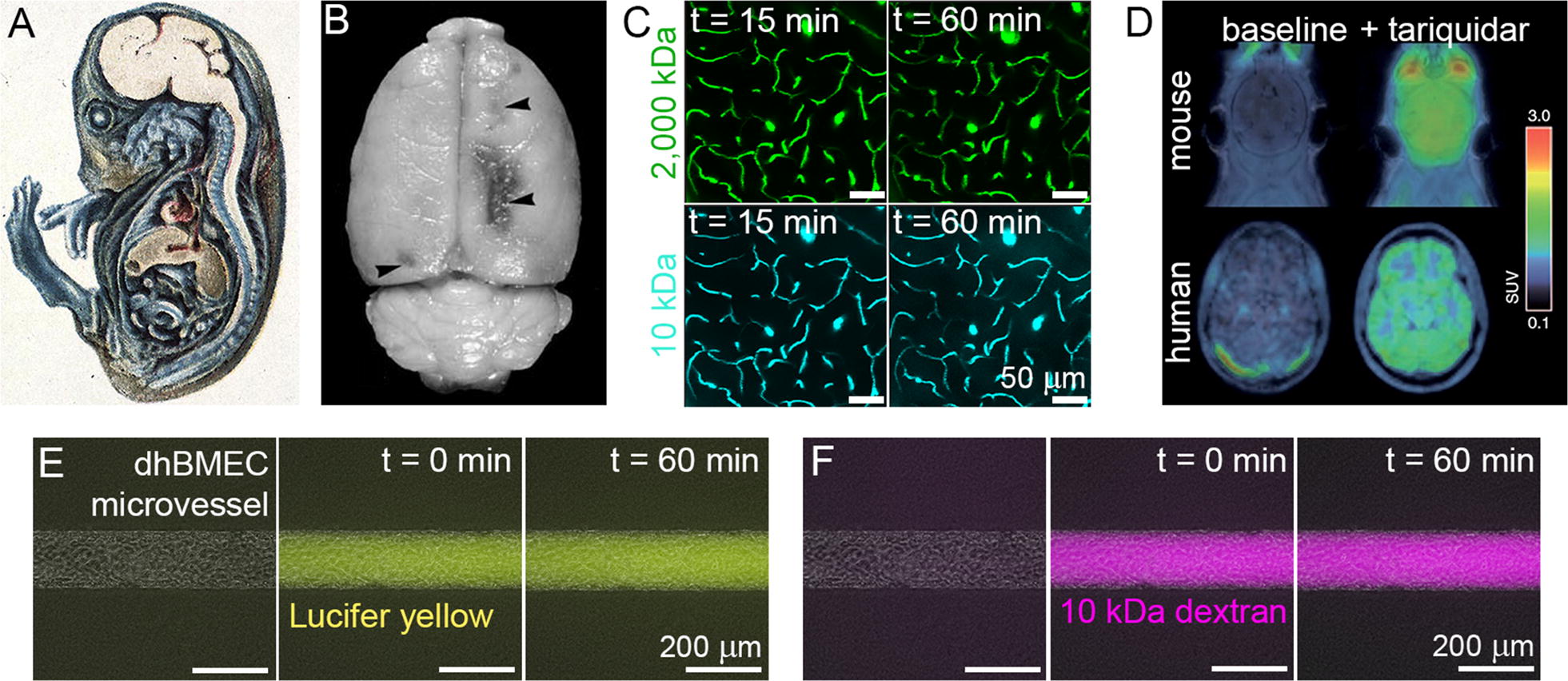
Blood–brain barrier permeability. **A** Guinea pig embryo injected with trypan blue demonstrates restriction of dye entry into CNS (from [135]). **B** Brain of a rat with chronic hypertension showing areas of Evans blue extravasation in the boundary zone areas (from [141]). **C** Adult mice injected with fluorescently-labeled dextrans (10 and 2000 kDa) and imaged with two-photon microscopy show lack of significant dye extravasation over 1 h (from [142]). **D** Positron emission tomography (PET) imaging of radiolabeled verapamil in mouse and human brains with and without p-glycoprotein inhibition using tariquidar. The color bar indicates brain-to-plasma ratio, a measure of drug penetration (from [143]). **E**, **F** Permeability experiments using Lucifer yellow and 10 kDa dextran in tissue-engineered iPSC-derived brain microvessels (from [24])

Defining quantitative benchmarks for barrier function is surprisingly difficult, and almost all quantitative in vivo data comes from animal models. The discovery of the BBB dates back to the observation by Paul Ehrlich that systemically injected Trypan blue stains all organs except the brain (Fig. 4A) [135, 136]. These experiments have subsequently been repeated with other dyes, such as Evans blue (961 Da) in many animal models (Fig. 4B, C) [135]. These dyes do not appreciably enter the brain as they are bound to albumin in circulation (67 kDa, ~ 10 nm in size). The in vivo permeability of the BBB to various solutes has been performed using various experimental protocols in animal models. The permeability of Lucifer yellow in 15 µm pial post-capillary venules in a rat model was reported to be 1–2 × 10^−7^ cm s^−1^ [137]. In zebrafish, where the cerebrovasculature is visible without surgical manipulation, 10 kDa dextran was not observed to enter the brain parenchyma [138]. However, in a series of experiments using multiphoton microscopy in 20–40 µm pial post-capillary venules in a rat model [139, 140], the permeability of sodium fluorescein (332 Da) was determined to be 1.46 × 10^−6^ cm s^−1^ and the permeability of 10 kDa dextran was 3.1 ± 1.3 × 10^−7^ cm s^−1^. While the permeability of the larger 10 kDa dextran is fivefold smaller than fluorescein, it is close to the permeability of the small molecule Lucifer yellow as described above [137]. Thus, there are discrepancies across in vivo studies in animal models that make it difficult to provide definitive values for benchmarking. Therefore, we provisionally suggest that for tissue-engineered models: (1) the permeability of Lucifer yellow should be ≤ 2 × 10^−7^ cm s^−1^, and (2) the permeability of 10 kDa dextran, solutes that bind to albumin, or other large molecules (e.g. albumin) should be negligible (≤ 1 × 10^−7^ cm s^−1^).

In vitro models of the BBB generally fail to achieve physiological permeability. In one of the very few studies of tissue-engineered BBB microvessels incorporating primary human BMECs, astrocytes, and pericytes, the permeability of 3 kDa dextran was reported to be 2–4 × 10^−6^ cm s^−1^ [43]. As acknowledged by the authors, this high permeability for a large molecule does not recapitulate BBB barrier function and is likely associated with low TEER values (40–50 Ω cm^2^) of the primary BMECs. In iPSC-derived human BMEC microvessels (with no other cell types), the permeability of Lucifer yellow was reported to be 2–3 × 10^−7^ cm s^−1^ (Fig. 4E) [24], close to values reported in a rat model [137]. In addition, the permeability of 10 kDa fluorescently labeled dextran was below the detection limit (Fig. 4F) [24]. TEER values for the iPSC-derived hBMECs were > 1500 Ω cm^2^, in the range thought to be physiological. Taken together these results further support the hypothesis that physiological TEER values are associated with negligible paracellular transport and permeabilities that are related to transcellular transport alone. A recent report of a self-organized human BBB microvascular network reported 10 kDa dextran permeability of ~ 2 × 10^−7^ cm s^−1^, matching values reported from multiphoton studies [45].

### Efflux transport

To regulate entry of small molecules into the brain by passive transport, BMECs express an array of efflux transporters, the most well-known of which are the P-glycoprotein (P-gp) and Breast Cancer Resistant Protein (BCRP) pumps [144]. These pumps are generally polarized on the luminal surface of BMECs and are capable of effluxing a wide range of chemically diverse compounds [145].

A common functional method to confirm the presence of efflux pumps is to perform permeability experiments in a Transwell^®^ assay, measuring both apical-to-basolateral (AB) and basolateral-to-apical (BA) permeability of a solute that is a substrate for the pump. If the efflux pump is polarized to the apical surface on the BMECs then the efflux ratio (ER) which is defined as P_BA_/P_AB_, is > 1. Due to the variations in experimental measurements, a molecule is generally considered to be an efflux substrate if ER > 2. Efflux ratios are typically in the range of 2–10, although higher values have been reported [146]. Confirmation that the ER is related to differences in efflux transport can be obtained by introducing an inhibitor. For complete inhibition of a solute that is only a substrate of the target efflux pump, the ER will decrease to 1.0. However, the inhibitor may not be completely effective in blocking efflux and/or efflux at other pumps may result in only a small decrease in ER.

In tissue-engineered models it is difficult to perform bi-directional permeability measurements and hence experiments generally rely on measurement of differences in permeability (lumen to matrix) with and without inhibitor. This method is most effective if the inhibitor is very efficient in blocking the target efflux pump (e.g. P-gp) and the solute is only a substrate for that efflux pump. A common approach is to measure the permeability of Rhodamine 123 which is a P-gp substrate and to use a P-gp inhibitor such as cyclosporin A or tariquidar to reduce efflux. An alternative approach is to use gene editing to delete or knock down an efflux pump to replicate results from genetically engineered mouse models, however, this method may result in secondary effects that also modulate barrier function. The presence of efflux pumps can be confirmed by immunohistochemistry, although it is difficult to quantify absolute expression levels or polarization to the luminal or abluminal membrane.

Studies of efflux in humans are rare. Continuous intravenous infusion of tariquidar, a P-gp inhibitor, increased the brain penetration of radio-labeled verapamil by 2.7-fold in healthy human subjects, a 60% reduction in P-gp activity (Fig. 4D) [147]. Verapamil is used to treat migraines, but is also used as a substrate and inhibitor of the P-gp pump. Dual knockout of P-gp and BCRP in a mouse model resulted in a 40-fold increase in CNS penetration of efflux substrates [148].

### Endothelial cell turnover

Under quiescent conditions, the net turnover rate of BMECs is expected to approach zero. The net turnover rate is the difference between the proliferation rate and the rate of cell loss. Results from thymidine labeling in mice suggest that the turnover rate of endothelial cells in the brain is about 0.04% h^−1^ [149], an order of magnitude or more lower than endothelial cells in other tissues [150, 151]. Two-photon microscopy studies in capillary beds in the motor and somatosensory cortex of mice show no change in either capillary segment diameter, capillary segment length, and the position of branch points over about 30 days [152, 153]. Assuming that these results imply no proliferation/loss over the imaging period, we estimate an upper limit of the net turnover rate of 0.001% h^−1^. In one of these studies, BrdU labeling of cortical microvessels in mice revealed no detectable endothelial cell division over 10 days during the post-natal period, indicating a cell division rate of zero during that time frame [153]. Additionally, formation and elimination of microvessel branch points decreased with age, resulting in no formation or elimination of microvessel branch points over 30 days in adult mice [153]. Turnover is an important but often overlooked parameter in benchmarking tissue engineered vascular models. Validation is complicated by the lack of physiological data for the dynamics of endothelial cell division and loss in vivo in humans or animal models. Based on intravital microscopy experiments we provisionally suggest that target values for cell division and cell death are ≤ 0.001% h^−1^, with a net turnover rate approaching zero.

### Astrocytes

**Fig. 5:**
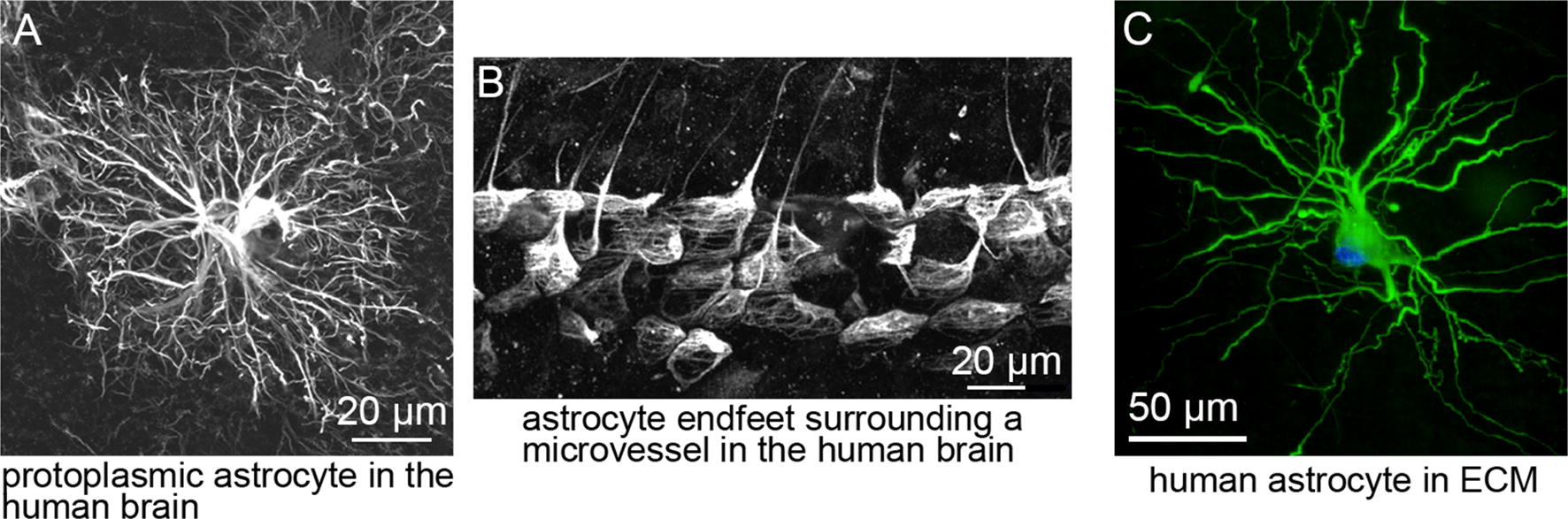
Astrocyte morphology. **A** Protoplasmic astrocyte in the human brain (adapted from [158]). **B** Astrocyte end-feet surrounding a microvessel in the human brain (adapted from [158]). **C** Astrocyte derived from neural progenitor cell cultured in an extracellular matrix consisting of collagen type I, Matrigel, and hyaluronic acid (from [170])

Astrocytes are involved in many processes in the brain, including neurotransmitter uptake and release, stress response, and neurovascular coupling [154–157]. Astrocytes typically have star-shaped morphologies with small cell bodies and radial branched processes that occupy distinct domains (Fig. 5A) [158]. These processes terminate in end-feet that completely ensheath capillaries (Fig. 5B) [157–159]. Human cortical astrocytes have a cell body approximately 10 µm in diameter, and extend ~ 40 primary process from the cell body resulting in an overall domain of about 150 µm [158]. In contrast, mouse astrocytes occupy domains of about 50 µm and extend fewer radial processes than their human counterparts [158].

In response to trauma or pathological tissue damage, astrocytes become activated, a process known as reactive gliosis [160–162]. Astrocyte activation is characterized by marked changes in protein expression [160, 162–164], a hallmark of which is the increased expression of intermediate filament proteins including glial fibrillary acidic protein (GFAP) and vimentin [160, 165]. Astrocytes also secrete a wide range of soluble growth factors (such as bFGF and GDNF), inflammatory cytokines, and extracellular matrix proteins [160, 163].

GFAP expression is often used as an astrocyte-specific marker, even though it is primarily a marker of activated astrocytes. S100B is also widely used to identify astrocytes, although expression may be limited to certain astrocyte subsets [166]. Two markers associated with BMEC-astrocyte signaling are aquaporin 4 (AQP4) and the potassium ion channel, KiR4.1, which are highly expressed in astrocyte end-feet surrounding brain capillaries [167, 168].

In BBB research, astrocytes are usually cultured in 2D (e.g. in the basolateral chamber in a Transwell^®^ assay). Physiological morphology is not believed to be critical to these experiments since the purpose is simply to provide a source of soluble factors secreted by the astrocytes. In 2D culture, the morphology and number of processes emanated depends on the surface coating, and the cells generally express GFAP, characteristic of activation [169]. In 3D culture, the morphology and level of GFAP expression in human astrocytes is strongly dependent on the composition and mechanical properties of the matrix material [170]. Human astrocytes cultured in gels composed of collagen, HA, and Matrigel exhibited a highly branched morphology, typical of their morphology in vivo (Fig. 5C), and very low levels of GFAP expression, a hallmark of quiescent cells [170].

Suggested benchmarks for incorporation of astrocytes into BBB models: (1) small cell body with radial branched processes with a domain size of about 150 µm. (2) End-feet extending to capillaries or microvessels. (3) High expression of astrocyte markers KiR4.1 and AQP4. (4) Negligible expression of activation markers such as GFAP and vimentin under quiescent conditions.

### Pericytes

**Fig. 6:**
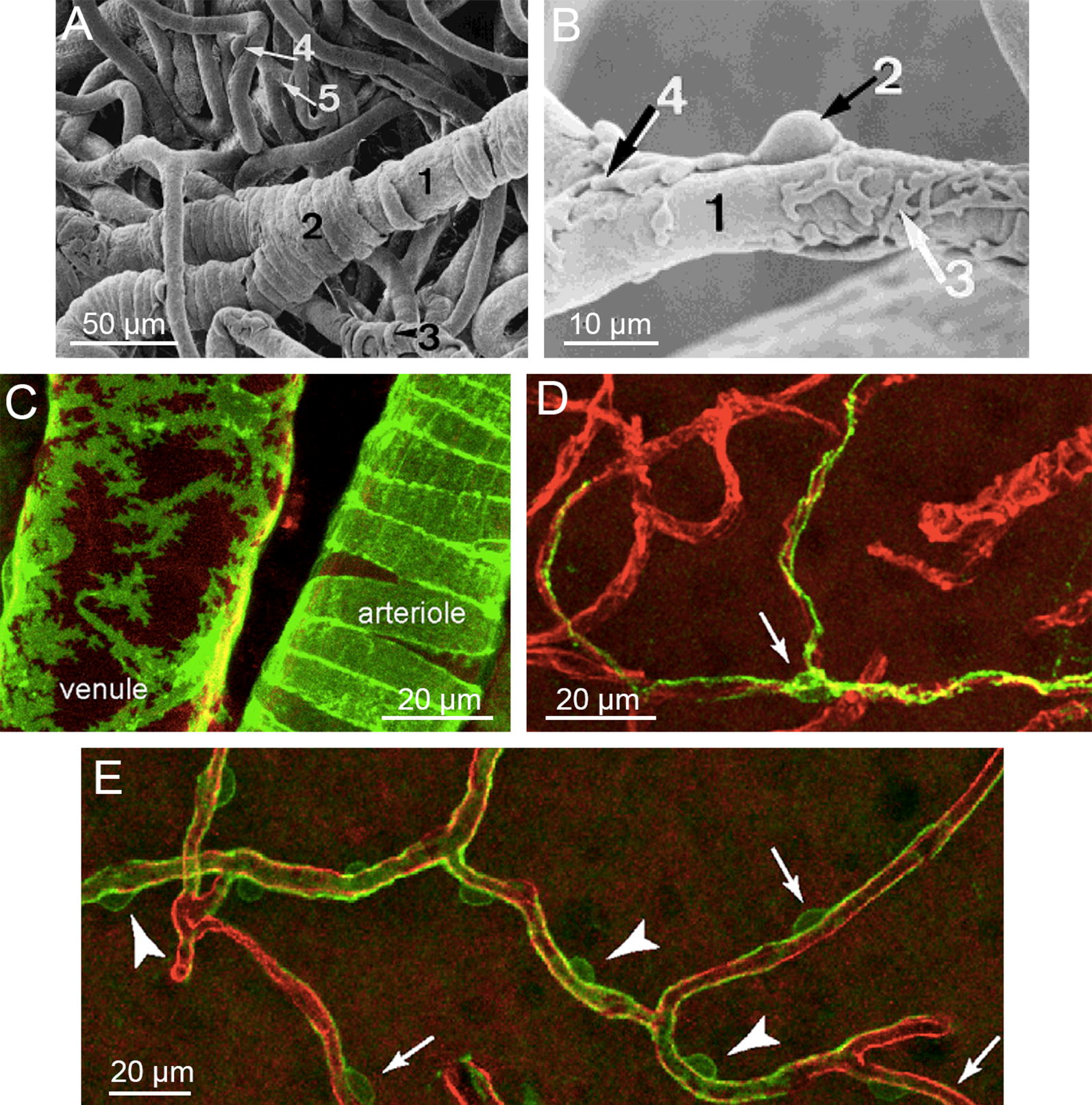
Perivascular cell morphology. **A** Scanning electron micrograph of a replica of an arteriole from human spinal cord showing: circumferentially wrapped VSMCs (2) and a capillary pericyte (4). **B** Pericyte-like structure (2) with primary and secondary processes (3). **C** Pericytes (green) and VSMCs (green) on venules and arterioles of NG2cre:mT/mG transgenic mice. **D** Thin-strand pericyte (green) extending long processes. Arrow denotes cell body. **E** Regions of transition from hybrid VSMC/pericyte morphology (arrowheads) to capillary pericyte morphology (arrows). **A**, **B** Adapted from [74]. **C**–**E** Adapted from [173]

Defining the morphology, organization, and function of pericytes in the brain has been inconsistent and controversial. Recent imaging studies in genetically engineered mouse models suggest that much of the confusion over pericyte function arises from the diversity of perivascular cell types in the cerebrovasculature [152, 171–174]. Perivascular cells surrounding arterioles express smooth muscle actin (αSMA) and are identified as vascular smooth muscle cells (VSMCs) [172, 173]. These cells extend processes around the circumference of the arterioles that appear as neighboring rings or bands up to 7 µm wide around microvessels (Fig. 6A, C).

In capillaries, perivascular cells are sparse and express platelet derived growth factor receptor (PDGFRβ), the proteoglycan neural/glial antigen 2 (NG2), and CD13 [171–173, 175, 176]. These cells are defined as pericytes, and are characterized by a bump-on-a-log morphology with an ovoid-shaped nucleus and narrow processes that are predominantly aligned along the capillary, and are completely surrounded by basement membrane (Figs. 1D, 6B, D, E) [152, 172–174]. The average density of pericytes in mouse models is about one cell per 80 µm length of capillary [152]. The ratio of pericytes to endothelial cells has been reported at approximately 1:5 by electron microscopy analysis of a small sample of rat cerebral capillaries [177]. In post-capillary venules, the perivascular cells, also identified as pericytes, extend both longitudinal and circumferential processes (Fig. 6C).

The VSMCs that surround arterioles are contractile and are believed to contribute to neurovascular coupling [172, 173], however, the role of capillary pericytes in blood flow regulation remains to be established [178, 179]. A further complication is that perivascular cells in the transition region from arterioles to capillaries express intermediate levels of αSMA and are difficult to classify as either VSMCs or pericytes.

In cell culture, pericytes are multipotent, with the capability to be differentiated into mesenchymal cells and neural cells [180–183]. This discovery led to the hypothesis that pericytes may be a source of other brain-specific cell types and play a role in stress and injury response in the brain [180]. However, this remains to be confirmed since recent evidence suggests that pericytes maintain their identity during normal aging or in pathological settings [184], and hence they are multipotent only after in vitro culture, and not in vivo.

Perivascular cells play a major role in angiogenesis where activated endothelial cells recruit pericytes through secretion of PDGF-ΒΒ which binds to PDGFRβ with high affinity. PDGF-ΒΒ is localized to the endothelium through binding to heparin sulfate proteoglycans (HSPGs). The role of pericytes in angiogenesis is largely derived from studies of neovascularization associated with diseases such as cancer [185], and hence the response of pericytes to disease- or trauma-induced angiogenesis remains to be elucidated. In a microfluidic model of angiogenesis involving microvessels formed from human umbilical vein endothelial cells, co-culture resulted in recruitment of human placental pericytes to sprouts and refinement of the nascent vasculature [186].

Suggested benchmarks for incorporation of pericytes into BBB models: (1) pericytes are located on the abluminal surface of models of capillaries or post-capillary venules. (2) Pericytes exhibit bump-on-a-log morphology with predominantly longitudinally aligned processes. (3) Pericytes express PDGFRβ, NG2, and CD13; do not express αSMA. (4) Pericytes are embedded within basement membrane.

### Astrocytes, pericytes, and barrier function

**Fig. 7:**
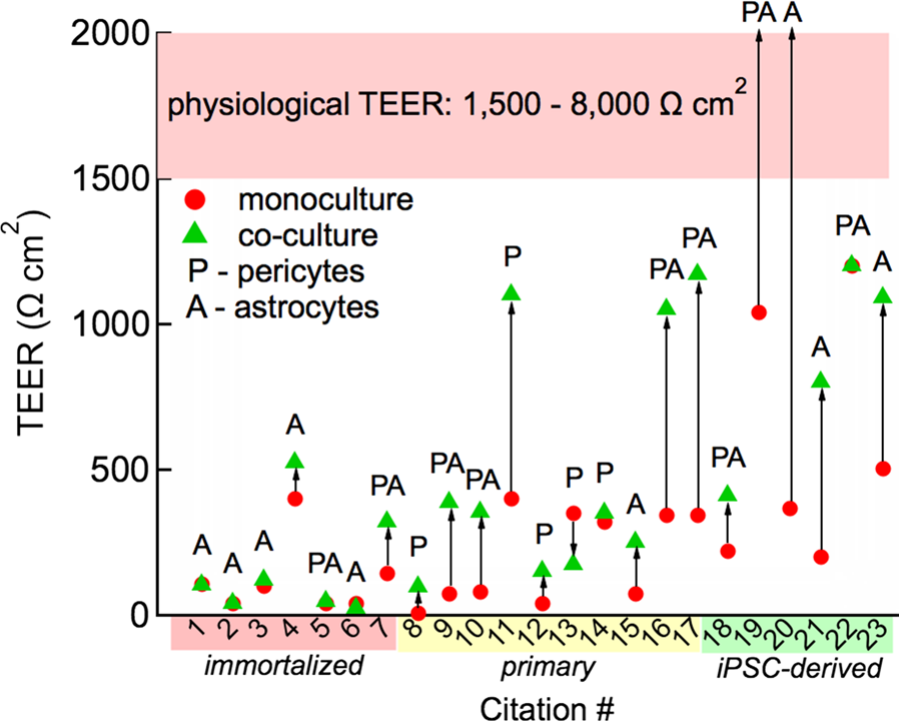
Compilation of changes of TEER values for BMEC monolayers with or without astrocytes or astrocyte-conditioned media (A), or pericytes (P) in the basolateral chamber or on the basolateral side of the Transwell^®^ membrane. Note that a wide range of primary, immortalized, and neural progenitor cell-derived cells from multiple species have been classified as either astrocytes or pericytes for simplicity, but individual references should be examined for cell sourcing details. Key for citations: 1—[196], 2—[197], 3—[194], 4—[191], 5—[195], 6—[132], 7—[17], 8—[188], 9—[190], 10—[189], 11—[193], 12—[187], 13—[198], 14—[199], 15—[200], 16 and 17 [201], 18—[1], 19—[2], 20—[17], 21—[15], 22—[9], 23—[21]

Numerous in vitro studies have reported that TEER of endothelial monolayers is increased in the presence of astrocytes, astrocyte extract, or pericytes, and this trend is cited as evidence of the role of astrocytes and pericytes in upregulation and maintenance of barrier function in vivo [187–195]. However, in many of these experiments, the final TEER values are still well below physiological (1500–8000 Ω cm^2^), casting some doubt on the in vivo relevance of these increases (Fig. 7). For example, the TEER of primary murine BMECs increased from about 35 Ω cm^2^ to about 140 Ω cm^2^ with pericytes in the basolateral chamber [187]. As described previously, monolayers of iPSC-derived hBMECs exhibit TEER values in the physiological range, particularly when derived with retinoic acid [2, 4], suggesting that astrocytes and pericytes are not essential for achieving physiological TEER values in in vitro models, provided they are cultured in the presence of key exogenous factors. However, monolayers of iPSC-derived hBMECs cultured under conditions with sub-physiological TEER values have approached [15, 21] or attained [2, 17] physiological values when co-cultured with astrocytes and/or pericytes (Fig. 7). Taken together, these results suggest that astrocytes and pericytes are not responsible for establishing barrier function, but can secrete factors that promote recovery or repair. If this hypothesis is correct, then we would expect that co-culture of astrocytes and/or pericytes with optimally differentiated hBMECs would have no effect on barrier tightness, but may aid in recovery of barrier function in response to simulation of injury or stress. Whether these results from in vitro models recapitulate the roles of these cells in the neurovascular unit remains to be established.

## Summary

Here we describe 12 parameters for benchmarking in vitro BBB models. These parameters are associated with structure (ultrastructure, wall shear stress, geometry), microenvironment (basement membrane and extracellular matrix), barrier function (TEER, permeability, efflux transport), cell function (expression of BBB markers, turnover), and co-culture with other cell types (astrocytes and pericytes). The relevant benchmarks are dependent on the purpose of the model and provide a starting point to guide validation and future developments.

Although the complexity of tissue-engineered BBB models has advanced significantly in recent years, incorporation of multiple characteristics of the BBB remains challenging. Nonetheless, recent advances in tissue engineering and stem cell technology provide the foundation for new frontiers in BBB modeling. (1) Improved visualization of barrier function and turnover: quantifying barrier function and turnover in vivo is difficult and hence new models that enable imaging of these functions in real-time would increase understanding of BMECs in health and disease [202]. (2) Hierarchical BBB models comprised of an arteriole, capillary bed, and venule. Templating approaches generally mimic the structure of post-capillary venules, while self-organization approaches mimic brain capillaries. Recent advances in transcriptomics have precisely mapped zonation of the brain vasculature [203], providing a basis for comparison. Additionally, novel techniques to generate capillaries based on sprouting angiogenesis or growth along patterned channels may support this aim [35, 204]. (3) Recapitulating neurovascular coupling: neuronal activity and blood supply are matched via changes in arteriole and capillary tone mediated by signaling between endothelial cells, pericytes, astrocytes and neurons. Incorporation of neurons into BBB-on-a-chip models that are responsive to neuronal activity would represent a significant advance. Recently, neurometabolic coupling was demonstrate in vitro [205]. (4) Recapitulating human disease: complex microfluidic models of brain disease typically lack endothelial cells; for example, recent work demonstrated microfluidic triculture of neurons, astrocytes and microglia to mimic Alzheimer’s disease [206]. Integration of microfluidic models of brain disease and of the BBB models will support studies of human disease with increasing fidelity.
